# Soluble aggregates of the amyloid-β peptide are trapped by serum albumin to enhance amyloid-β activation of endothelial cells

**DOI:** 10.1186/1754-1611-3-5

**Published:** 2009-04-27

**Authors:** Adriana A Reyes Barcelo, Francisco J Gonzalez-Velasquez, Melissa A Moss

**Affiliations:** 1 Department of Chemical Engineering, University of South Carolina, 2C02 Swearingen Engineering Center, Columbia, South Carolina 29208, USA; 2 Kraft Foods, Inc, 200 Deforest Dr, East Hanover, NJ 07936, USA

## Abstract

**Background:**

Self-assembly of the amyloid-β peptide (Aβ) has been implicated in the pathogenesis of Alzheimer's disease (AD). As a result, synthetic molecules capable of inhibiting Aβ self-assembly could serve as therapeutic agents and endogenous molecules that modulate Aβ self-assembly may influence disease progression. However, increasing evidence implicating a principal pathogenic role for small soluble Aβ aggregates warns that inhibition at intermediate stages of Aβ self-assembly may prove detrimental. Here, we explore the inhibition of Aβ_1–40 _self-assembly by serum albumin, the most abundant plasma protein, and the influence of this inhibition on Aβ_1–40 _activation of endothelial cells for monocyte adhesion.

**Results:**

It is demonstrated that serum albumin is capable of inhibiting in a dose-dependent manner both the formation of Aβ_1–40 _aggregates from monomeric peptide and the ongoing growth of Aβ_1–40 _fibrils. Inhibition of fibrillar Aβ_1–40 _aggregate growth is observed at substoichiometric concentrations, suggesting that serum albumin recognizes aggregated forms of the peptide to prevent monomer addition. Inhibition of Aβ_1–40 _monomer aggregation is observed down to stoichiometric ratios with partial inhibition leading to an increase in the population of small soluble aggregates. Such partial inhibition of Aβ_1–40 _aggregation leads to an increase in the ability of resulting aggregates to activate endothelial cells for adhesion of monocytes. In contrast, Aβ_1–40 _activation of endothelial cells for monocyte adhesion is reduced when more complete inhibition is observed.

**Conclusion:**

These results demonstrate that inhibitors of Aβ self-assembly have the potential to trap small soluble aggregates resulting in an elevation rather than a reduction of cellular responses. These findings provide further support that small soluble aggregates possess high levels of physiological activity and underscore the importance of resolving the effect of Aβ aggregation inhibitors on aggregate size.

## Background

Alzheimer's disease (AD) is the leading cause of dementia in the elderly, afflicting a new victim every 71 seconds [1]. AD is characterized by the accumulation of amyloid plaques in the brain parenchyma and cerebral microvasculature. These plaques are comprised primarily of fibrils formed via self-association of the amyloid-β peptide (Aβ). The assembly of monomeric Aβ into fibrillar form has been implicated in the pathogenesis of AD, a premise formally set forth in the amyloid hypothesis [2,3]. In particular, genetic mutations associated with early onset AD promote Aβ assembly by either elevating total Aβ production or increasing the relative amount of the longer, more fibrillogenic form of the peptide. Overexpression of these mutations in transgenic animal models results in an age-dependent development of amyloid plaques as well as deficits in reference and working memory [4]. Consequently, inhibition of Aβ self-assembly is under investigation as a therapeutic strategy for AD. Similarly, endogenous molecules that are able to regulate Aβ assembly can impact disease progression.

Recent revisions to the amyloid hypothesis implicate a principal role for soluble Aβ aggregates, including Aβ-derived diffusible ligands (ADDLs), oligomers, and protofibrils, in the pathogenesis of AD. Soluble Aβ aggregates are capable of eliciting a number of responses in neuronal cell systems, including the impairment of long-term potentiation, the initiation of synaptic loss, and the alteration of memory function [reviewed in [2,5]]. In addition, soluble Aβ aggregates have been demonstrated to selectively elicit changes in brain endothelial cells associated with the increased immune response observed in AD brain, including the activation of endothelial monolayers for increased adhesion and subsequent transmigration of monocyte cells [6] and the stimulation of increases in endothelial monolayer permeability [7]. Here, a specific role for small soluble aggregates was implicated by an inverse relationship between endothelial response and aggregate size.

Plasma proteins have the potential to mediate AD-linked inflammatory responses observed in the brain endothelium. A number of plasma proteins are known to bind various isoforms of Aβ, including Aβ_1–40 _and Aβ_1–42 _[reviewed in [8]]. In fact, > 95% of circulating Aβ is bound by carrier proteins in blood plasma [9,10], with the majority of Aβ bound to serum albumin [10]. Several plasma proteins, including serum albumin, are also associated with amyloid plaques deposited in the brain [reviewed in [11]].

Interactions between Aβ and plasma proteins have been observed to inhibit Aβ assembly [reviewed in [8]]. Sixty percent of the inhibitory activity present within human plasma has been ascribed to serum albumin [12], and this inhibitory activity has been suggested to account for the lack of Aβ fibril deposition in the periphery [12,13]. Serum albumin has been shown to inhibit the aggregation of Aβ_1–40 _and Aβ_1–42 _monomer [12] as well as the incorporation of monomeric peptide into Aβ_1–40 _and Aβ_1–42 _fibrils [12], Aβ_12–28 _oligomers [13], and tissue sections isolated from AD brain [12]. Some studies suggest that serum albumin preferentially binds small Aβ aggregates [12,13]. Trapping these small soluble aggregates has the potential to increase Aβ stimulation of brain endothelium.

In the current study, we examine the relationship between the *in vitro *inhibitory activity of serum albumin and the activation of endothelial monolayers by inhibited Aβ_1–40 _aggregate preparations. An inhibitory role for bovine serum albumin (BSA) in both the formation and subsequent growth of Aβ_1–40 _aggregates is confirmed and shown to be dose-dependent. The stoichiometry observed for BSA inhibition of aggregate growth suggests that BSA is capable of binding Aβ_1–40 _aggregates. Furthermore, the characterization of Aβ_1–40 _aggregates via dynamic light scattering (DLS) demonstrates that BSA inhibition of Aβ_1–40 _aggregate formation from monomeric peptide can increase populations of small soluble aggregate species. Inhibition of Aβ_1–40 _aggregate formation from monomeric peptide is not paralleled by a dose-dependent decrease in Aβ-induced activation of endothelial cells for monocyte adhesion. Instead, endothelial monolayer activation is enhanced when intermediate levels of inhibitor activity lead to an increase in the number of small soluble Aβ_1–40 _aggregates. These results demonstrate that soluble Aβ aggregates trapped by inhibitors of Aβ self-assembly can enhance physiological responses associated with AD.

## Methods

### Materials

Aβ_1–40 _was purchased from AnaSpec (San Jose, CA) or W. M. Keck Biotechnology Resource Laboratory (New Haven, CT). BSA was obtained from EMD Chemicals (Gibbstown, NJ). Thioflavin T, RPMI 1640 medium, fetal bovine serum (FBS), hydrocortisone, gelatin, and Calcein-AM were obtained from Sigma (St. Louis, MO). CS-C Medium and other CS-C culture reagents were obtained from Cell Systems (Kirkland, WA). Collagen was obtained from PureCol INAMED (Fremont, CA).

### Preparation of Aβ_1–40 _monomer and fibril

Lyophilized Aβ_1–40 _was stored desiccated at -20°C until preparation of monomer or fibril as described previously [6]. Briefly, Aβ_1–40 _was reconstituted (2 mg/ml) in 50 mM NaOH and pre-existing aggregates were removed by size exclusion chromatography on a Superdex 75 HR10/30 column (GE Healthcare, Piscataway, NJ) equilibrated in 40 mM Tris-HCl (pH 8.0). Isolated monomer was used fresh or stored at 4°C for up to 24 h. To prepare fibrils, isolated monomeric Aβ_1–40 _(100–200 μM) was agitated vigorously at 25°C in the presence of 250 mM NaCl and 40 mM Tris-HCl (pH 8.0) for 24–30 h. Fibrils were evaluated via thioflavin T fluorescence, isolated via centrifugation (15 min, 18,000 × *g*), resuspended in 40 mM Tris-HCl (pH 8.0), and stored at 4°C for up to 1 week.

### Detection of Aβ_1–40 _aggregates using thioflavin T

As described previously [6], thioflavin T fluorescence was monitored for samples containing 10 μM thioflavin T solution in 40 mM Tris-HCl (pH 8.0). Fluorescence was measured on an LS-45 luminescence spectrometer (Perkin-Elmer, Waltham, MA) with excitation at 450 nm. Fluorescence (F) was taken as the integrated area under the emission curve (470–500 nm) with baseline (thioflavin T, BSA) subtraction.

### Aβ_1–40 _monomer aggregation assay

Aβ_1–40 _monomer was diluted to 20 μM in 40 mM Tris-HCl (pH 8.0) containing 150 mM NaCl and incubated in the absence (control) or presence of 20–80 μM BSA at 25°C and under vigorous agitation (vortex, 500 rpm). Reaction progress was monitored via thioflavin T fluorescence following dilution of an aliquot into 10 μM thioflavin T, and results are reported as the change in thioflavin T fluorescence (ΔF) with time. Subsequent to the final fluorescence measurement, aggregate size was assessed for undiluted samples via hydrodynamic radius (*R*_H_) measurements. Experiments in which thioflavin T fluorescence and *R*_H _were measured for BSA subjected to aggregation conditions in the absence of Aβ_1–40 _monomer served as a negative control and ascertained the negligible contribution of BSA to these signals.

### Determination of Aβ_1–40 _aggregate size using DLS

As described previously [6], aggregate size was assessed via *R*_H _measurements using a DynaPro MSX DLS instrument (Wyatt Technology, Santa Barbara, CA) to assimilate auto-correlated light intensity data for calculation of translational diffusion coefficients that could be converted to *R*_H _using the Stokes-Einstein equation. *R*_H _was assessed from the intensity-weighted histogram calculated via data regulation with Dynamics software (Wyatt Technology).

### Aβ_1–40 _fibril elongation assay

Aβ_1–40 _fibrils were diluted in 40 mM Tris-HCl (pH 8.0) containing thioflavin T and incubated for 15 min in the absence (control) or presence of BSA. To initiate growth, Aβ_1–40 _monomer was added for final concentrations of 40 μM monomer, 2 μM fibril, 0–10 μM BSA, and 10 μM thioflavin T. Reactions were incubated at 25°C without agitation, and the incorporation of monomer into growing fibrils was monitored via thioflavin T fluorescence. Experiments in which thioflavin T fluorescence of 40 μM Aβ_1–40 _monomer was monitored in the absence of fibrils confirmed that monomer self-assembly did not occur under these experimental conditions. In addition, experiments in which thioflavin T fluorescence of 2 μM Aβ_1–40 _fibrils was monitored without addition of monomer served as negative controls and reflected the stability of fibrils. Results are reported as the change in thioflavin T fluorescence (ΔF) with time, and elongation rates were determined by linear regression of this data.

### Cell culture

ACBRI 376 primary human brain microvascular endothelial cells (HBMVECs) (Cell Systems, Kirkland, WA) and the human pre-monocytic cell line THP-1 (American Type Culture Collection, Rockville, MD) were maintained and prepared for experiments as described previously [6]. Endothelial cells (passage 4–8) were seeded (5 × 10^5^–6 × 10^5 ^cells/ml) onto coated (2.0% gelatin, 100 μg/ml collagen) 96-well flat-bottom culture plates and sustained until confluence in CS-C Serum Free Medium supplemented with hydrocortisone (550 nM). THP-1 cells maintained in supplemented RPMI 1640 medium containing 10% FBS were fluorescently labelled for adhesion experiments via incubation with Calcein-AM followed by resuspension in Dulbecco's PBS (D-PBS) (2 × 10^5 ^cells/ml).

### Adhesion assay

Confluent HBMVEC monolayers were treated for 24 h with Aβ_1–40 _monomer aggregated in the absence (positive control) or presence of BSA. Reaction products were diluted into culture medium containing hydrocortisone for a final Aβ_1–40 _concentration of 5 μM. Parallel treatment with an equivalent dilution of BSA or buffer served as negative controls. As described previously for adhesion assays [6], activated monolayers were washed and incubated with Calcein-labelled THP-1 cells (2 × 10^4^cells/well) for 30 min (37°C, 5% CO_2_). Nonadherent cells were removed by gentle washing (D-PBS). The number of adherent cells was assessed by Calcein fluorescence employing a Synergy 2 microplate reader (BioTek Instruments, Inc., Winooski, VT) equipped with an excitation filter of 485 ± 10 nm and an emission filter of 528 ± 10 nm and using baseline (D-PBS) subtraction. Results are reported as the percentage of adherent cells [(100%)·(Cells_adherent_/Cells_initial_)].

### Statistical analysis

Statistical analysis was performed with a one-way ANOVA using GraphPad Prism 5 software (San Diego, CA). Dunnett's test was used for multiple comparisons. *P *< 0.05 was considered significant. Linear regression was assessed using the coefficient of determination, r^2^.

## Results

### BSA inhibits aggregation of Aβ_1–40 _monomer

To assess the effect of BSA on Aβ_1–40 _fibril assembly from monomeric peptide, monomer aggregation was induced by continuous agitation in the presence of ratios of BSA to Aβ_1–40 _monomer ranging from equimolar to 4-fold excess. The formation of aggregated β-sheet structure was monitored using thioflavin T fluorescence. To ensure that the presence of BSA did not impede detection of Aβ aggregates, it was confirmed that the thioflavin T fluorescence of pre-formed Aβ_1–40 _fibrils was not reduced in the presence of BSA. These results indicate that BSA does not compete with thioflavin T for binding to fibrils nor does it sequester thioflavin T to prevent its binding to fibrils.

**Figure 1:**
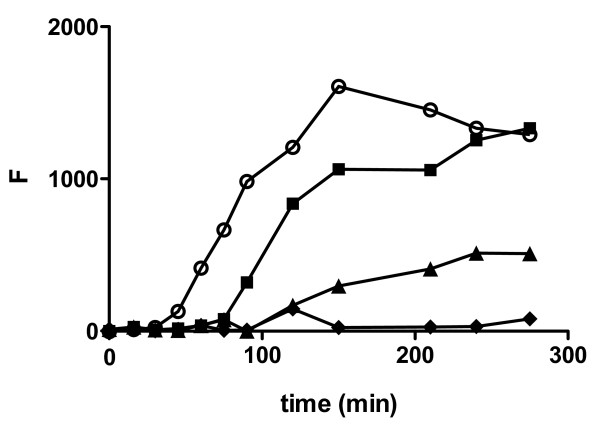
**Effect of BSA on Aβ_1–40 _monomer aggregation**. Aβ_1–40 _monomer was diluted to 20 μM in buffer containing 150 mM NaCl and 40 mM Tris-HCl (pH 8.0) and incubated alone (control, [white circle]) or in the presence of 20 μM [black square], 40 μM [black triangle], or 80 μM [black diamond] BSA. Aggregation of monomer was induced by continuous agitation and monitored using thioflavin T fluorescence. Results are representative of three independent experiments.

As shown in Figure 1, 20 μM Aβ_1–40 _monomer incubated in the absence of BSA exhibited the characteristic lag, indicative of nucleus formation, followed by rapid aggregate growth and concluding with a plateau as equilibrium was reached. BSA-induced changes in Aβ_1–40 _monomer aggregation were assessed by evaluating both extension of the lag time and reduction of the plateau fluorescence level. When BSA was present at equimolar concentrations with Aβ_1–40 _monomer, the lag time was nearly doubled (Figure 1, Table 1), suggesting that BSA can intervene at early points along the self-assembly pathway. As the concentration of BSA was increased to a level 2-fold in excess of Aβ_1–40 _monomer, the lag time was further extended by almost 2.5-fold and the equilibrium plateau was reduced, indicating that at higher concentrations BSA can reduce the quantity of aggregated Aβ. When Aβ_1–40 _monomer was agitated in the presence of a 4-fold excess of BSA, nearly complete inhibition was observed over the 4.5 h period. BSA subjected to aggregation conditions in the absence of Aβ_1–40 _monomer exhibited negligible change in thioflavin T fluorescence. These results illustrate the ability of BSA to abrogate Aβ_1–40 _monomer aggregation at concentrations in excess of Aβ_1–40 _monomer and further demonstrate the dose-dependent nature of this inhibition as BSA concentrations approach stoichiometric ratios with Aβ_1–40_.

**Table 1:** Dose-dependent inhibition of Aβ_1–40 _monomer aggregation and fibril growth in the presence of BSA ^a^

Reaction	Monomer Aggregation ^c^		Fibril Growth ^d^
(BSA:Aβ^b^)	Lag Time (fold increase)	Plateau (% reduction)	Growth Rate (% inhibition)
1:4	---	---	49 ± 11 *
1:1	1.9 ± 0.4	6 ± 6	61 ± 17 *
2:1	2.4 ± 0.6	34 ± 18	---
4:1	> 7 *	97 ± 2 *	---
5:1	---	---	80 ± 11 *

^a ^Parameters are expressed as mean ± SE, n = 3.

^b ^Aβ_1–40 _fibril concentrations are expressed in monomer units.

^c ^Monomer aggregation was measured as in Figure 1.

^d ^Fibril growth was measured as in Figure 2.

* p < 0.05 compared to control.

### BSA inhibits growth of Aβ_1–40 _fibrils via monomer addition

To determine whether BSA is capable of interacting with pre-formed Aβ_1–40 _aggregates to inhibit later stages of Aβ_1–40 _assembly, the growth of Aβ_1–40 _fibrils was assessed following pre-incubation in the presence of BSA at ratios of BSA to Aβ_1–40 _fibril ranging from substoichiometric to 5-fold excess, where fibril concentrations are expressed in monomer units. Fibril growth was induced via addition of Aβ_1–40 _monomer, and the incorporation of monomer into growing fibrils was monitored as the change in thioflavin T fluorescence. Thioflavin T may itself act as both an indicator and an inhibitor of Aβ self-assembly. However, at stoichiometric ratios that exceed those included within fibril elongation reactions thioflavin T has been observed to have no effect upon the formation of Aβ fibrils from monomeric peptide [14]. In addition, the absence of inhibitory activity by thioflavin T within the Aβ fibril elongation assay has been experimentally confirmed for the conditions employed (data not shown).

**Figure 2:**
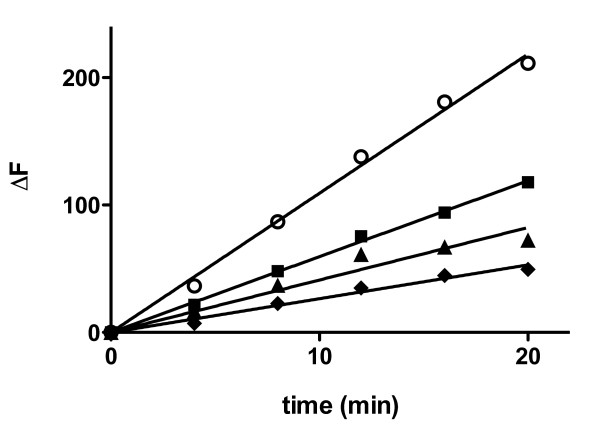
**Effect of BSA on Aβ_1–40 _fibril growth via monomer addition**. Aβ_1–40 _fibril in 40 mM Tris-HCl (pH 8.0) containing 10 μM thioflavin T was pre-incubated for 15 min alone or in the presence of BSA. Solutions were then diluted for final concentrations of 2 μM Aβ_1–40 _fibril (concentration expressed in monomer units) with 0 μM (control, [white circle]), 0.5 μM [black square], 2 μM [black triangle], or 10 μM [black diamond] BSA, and 40 μM Aβ_1–40 _monomer was added to induce fibril growth. Incorporation of Aβ_1–40 _monomer into fibrillar structures was monitored using thioflavin T fluorescence. Linear regression (solid lines) was performed to determine growth rates. Results are representative of three independent experiments.

As shown in Figure 2, steady growth was observed when 2 μM Aβ_1–40 _fibril was incubated with 40 μM Aβ_1–40 _monomer (Figure 2). This uninhibited rate of fibril growth was compared to the rate of growth observed in the presence of BSA to determine the extent of inhibition. Aβ_1–40 _fibrils pre-incubated in the presence of BSA at a level 5-fold in excess of monomeric units within Aβ_1–40 _fibrils exhibited a much slower rate of growth (Figure 2) and therefore significant inhibition (Table 1), confirming that BSA is able to prevent the addition of Aβ_1–40 _monomer to pre-formed Aβ_1–40 _fibrils. Pronounced inhibition was also evident when BSA was present at concentrations equimolar to monomeric units within Aβ_1–40 _fibrils. Further reduction in the concentration of BSA to a substoichiometric ratio of 1:4 BSA:Aβ_1–40 _fibril continued to inhibit fibril growth at lower but still significant levels. These results illustrate the ability of BSA to modulate the growth of pre-formed Aβ_1–40 _aggregates in a dose-dependent manner and, in contrast to BSA inhibition of monomer aggregation, demonstrate the strength of this inhibition at substoichiometic BSA concentrations.

### BSA inhibition of Aβ_1–40 _aggregate assembly modulates physiological activity

To assess the effect of BSA upon the physiological activity of Aβ_1–40 _aggregates, brain endothelial cells, which are in direct and continuous contact with circulating serum proteins, were selected. Aβ_1–40 _has been previously observed to stimulate HBMVECs for increased adhesion of both THP-1 monocytes [6,15,16] and primary human peripheral blood monocytes [6,16] via a mechanism that involves Aβ recognition of the receptor for advanced glycation end products [15,16]. The ability of BSA to attenuate this Aβ-induced adhesion in HBMVECs was explored.

**Figure 3:**
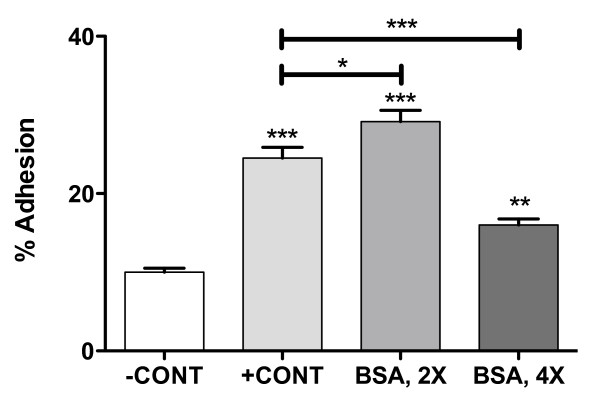
**Effect of BSA inhibition of Aβ_1–40 _monomer aggregation on endothelial adhesion in HBMVECs**. HBMVEC monolayers grown to confluence in 96-well plates were stimulated via 24 h incubation. Confluent monolayers were incubated alone (-CONT) or with 5 μM Aβ_1–40 _aggregates formed in the absence (+CONT) or presence of BSA at concentrations 2-fold (BSA, 2X) or 4-fold (BSA, 4X) in excess of Aβ_1–40 _monomer. Activated monolayers were washed and adhesion of Calcein-labelled THP-1 cells (2 × 10^4 ^cells/well) was assessed via fluorescence as described in Materials and Methods. * p < 0.05, ** p < 0.01, *** p < 0.001. Results are representative of three independent experiments performed with six repetitions. Error bars represent SEM.

When HBMVEC monolayers were treated with 5 μM Aβ_1–40 _aggregates formed in the absence of BSA, a 2.3-fold increase in adhesion of THP-1 monocytes relative to untreated control monolayers was observed (Figure 3). This result is similar to that reported previously [6]. Aβ_1–40 _aggregates formed in the presence of a 2-fold excess of BSA stimulated an even more pronounced 2.9-fold increase in adhesion of THP-1 monocytes, despite the decreased thioflavin T fluorescence observed under these aggregation conditions (Figure 1). In contrast, when Aβ_1–40 _aggregates were formed in the presence of a 4-fold excess of BSA, a smaller increase in THP-1 adhesion of only 1.5-fold was observed. BSA treatment of endothelial monolayers in the absence of Aβ made an insignificant contribution to the observed changes in adhesion. These results demonstrate that inhibition of *in vitro *Aβ_1–40 _monomer aggregation by BSA is not paralleled by a dose-dependent decrease in physiological activity.

### Physiological activity of Aβ_1–40 _aggregates correlates with aggregate size

Previous studies revealed that the size of Aβ_1–40 _aggregates determines their ability to stimulate endothelial monolayers for monocyte adhesion. The most pronounced increases in adhesion were observed for small soluble aggregates exhibiting *R*_H _values of 20–40 nm. Physiological activity decreased as aggregate size increased to 100 nm, and aggregates exceeding 100 nm increased endothelial adhesion only modestly [6]. A similar inverse correlation was also observed for Aβ_1–40 _stimulation of endothelial permeability [7]. Thus, it was speculated that the differential effect of BSA on the physiological activity of Aβ_1–40 _aggregate preparations might be related to differences in the resulting aggregate size.

**Figure 4:**
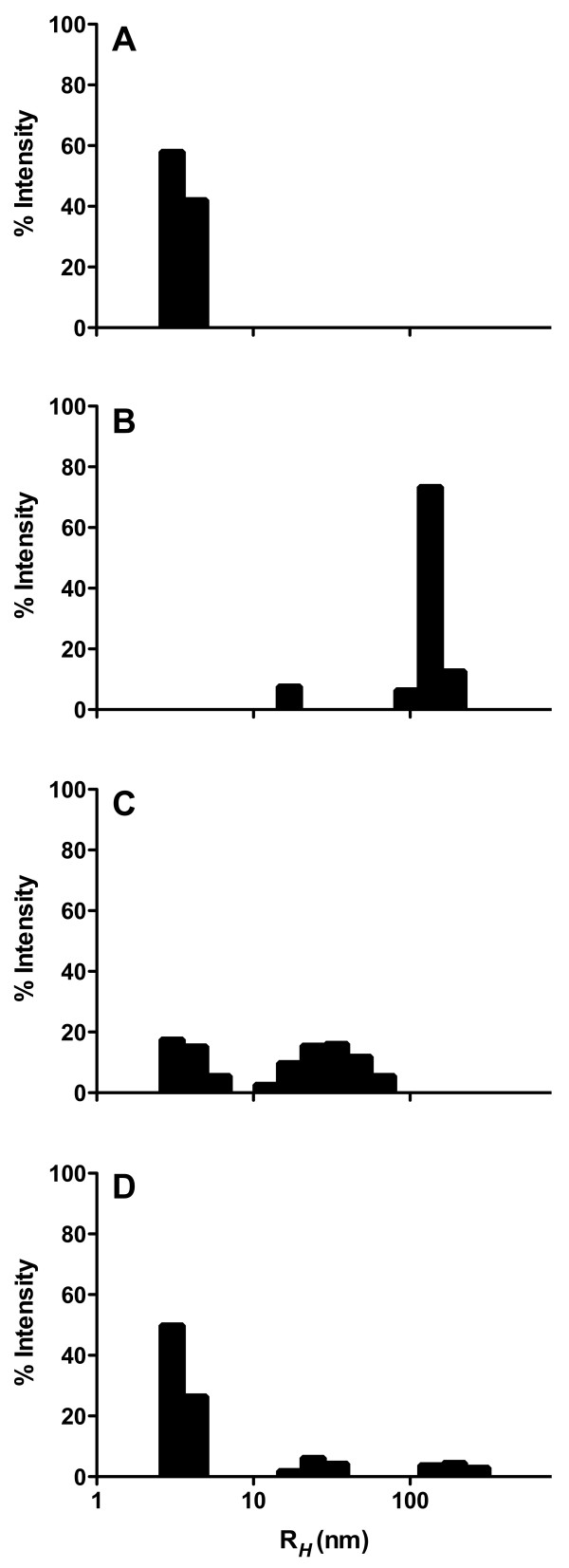
**Effect of BSA inhibition of Aβ_1–40 _monomer aggregation on aggregate size distribution**. Aβ_1–40 _monomer aggregations were performed as in Figure 1 for 20 μM Aβ_1–40 _monomer in the presence of 0 μM (panel B, control), 40 μM (panel C), or 80 μM (panel D) BSA. In addition, 80 μM BSA was subjected to aggregation conditions in the absence of Aβ_1–40 _monomer (panel A). DLS was employed to determine *R*_H _distributions. Results are presented as intensity-weighted histograms derived from data regularization with Dynamics Software (Wyatt Technology). Results are representative of two independent experiments.

DLS was employed to evaluate *R*_H _for aggregates formed in the presence and absence of BSA as well as BSA subjected to aggregation conditions in the absence of Aβ_1–40 _monomer. Aβ_1–40 _monomer incubated in the absence of BSA yielded aggregates with an *R*_H _of 140 nm (Figure 4B) that produced, as expected, a moderate but pronounced increase in endothelial adhesion (Figure 3). While addition of BSA at a concentration 2-fold in excess of Aβ_1–40 _monomer yielded less aggregated peptide (Figure 1), these aggregates exhibited a smaller *R*_H _of 34 nm (Figure 4C) and were thus capable of eliciting higher physiological activity (Figure 3). In contrast, Aβ_1–40 _aggregations performed in the presence of a 4-fold excess of BSA yielded little aggregated peptide (Figure 1) and exhibited a hydrodynamic radius of 3.4 nm (Figure 4D). This peak may be ascribed to BSA, as an identical peak was observed when BSA was subjected to aggregation conditions in the absence of Aβ_1–40 _monomer (Figure 4A). Due to the exponential relationship between scattered light and aggregate size, this BSA peak would obscure detection of Aβ monomer or aggregates exhibiting *R*_H _smaller than 3.4 nm. Thus, it may be deduced that following aggregation in the presence of a 4-fold excess of BSA, Aβ_1–40 _exists primarily as monomeric or oligomeric structures. However, a lower intensity peak at 22 nm (Figure 4D) indicates the presence of a small population of soluble aggregates with high physiological activity, which likely accounts for the modest increase in adhesion observed for this Aβ_1–40 _aggregate preparation (Figure 3). Together, these results demonstrate that the enhanced physiological activity observed for partially inhibited monomer aggregations correlates with an increase in the population of small soluble Aβ_1–40 _aggregates.

## Discussion

Aβ has the opportunity to interact *in vivo *with endogenous proteins, peptides, and small molecules prior to and during its self-assembly to form fibrillar aggregates which deposit in the brain parenchyma and cerebral microvasculature and become hallmarks of AD. Implications that small soluble aggregation intermediates may play a principal role in disease progression leave open the possibility that endogenous molecules which intervene at intermediate stages of self-assembly could elevate physiological activity. Several serum proteins, including serum albumin, have been observed to bind Aβ and inhibit the formation of fibrils [reviewed in [8]]. These proteins may influence Aβ activation of endothelial monolayers, a response which is specific for small soluble Aβ aggregates [6,17]. In the current study, we provide evidence that partial inhibition of Aβ_1–40 _aggregate assembly by BSA can enhance Aβ_1–40 _stimulation of endothelial monolayers for monocyte adhesion as a result of an increase in the number of small soluble aggregates.

Other studies have reported inhibition of Aβ aggregate assembly by serum albumin. Bohrmann et al. observed complete inhibition of Aβ_1–40 _monomer aggregation when human serum albumin (HSA) was present at an 11-fold excess [12] and a dose-dependent inhibition by both BSA and HSA for the incorporation of Aβ_1–40 _or Aβ_1–42 _monomer into immobilized fibrils [12]. Using solution NMR to facilitate atomic resolution of Aβ monomer and oligomer, Milojevic et al. demonstrated that the presence of HSA retarded the addition of monomeric Aβ_12–28 _to high molecular weight oligomers [13]. In the current study, dose-dependent BSA inhibition of both the formation of Aβ_1–40 _aggregates from monomeric peptide and the growth of fibrillar Aβ_1–40 _aggregates was examined to further understand the stoichiometry of these interactions. The decreasing inhibitory action observed in monomer aggregation assays as albumin reached a 1:1 ratio with monomeric Aβ_1–40 _(Figure 1, Table 1) indicates that BSA may interact with either Aβ_1–40 _monomer or oligomers containing a small number of monomeric subunits. Some studies have reported that HSA binds Aβ_1–40 _and Aβ_1–42 _at a 1:1 ratio [9,18] with K_d _of 5–13.5 μM [9,18] and 10.5 μM [9,18], respectively, suggesting that serum albumin may recognize monomeric Aβ. However, other studies have reported that BSA and HSA are incapable of binding monomeric Aβ_1–40 _and Aβ_1–28 _but instead recognize aggregated forms of the peptide [12,13], including oligomers [13].

The observed inhibition of Aβ_1–40 _fibril growth by monomer addition could likewise result either from the trapping of monomeric Aβ_1–40 _by BSA or from the blocking of sites for monomer addition via binding of BSA to Aβ_1–40 _fibrils. A dominant contribution of the latter mechanism is implicated by the pronounced inhibition of monomer addition to fibrils when Aβ_1–40 _monomer concentrations (40 μM) significantly exceed BSA levels (0.5 μM). Furthermore, the inhibition of Aβ_1–40 _fibril growth at substoichiometric ratios of BSA to monomeric units within Aβ_1–40 _fibrils (Figure 2, Table 1) combined with the previously reported micromolar affinity of serum albumin for Aβ [9,18] suggests that the binding of BSA to individual monomer units within Aβ_1–40 _fibril structures is not required for the inhibition of Aβ_1–40 _fibril elongation. Instead, BSA may bind aggregates at selected sites, among which are those that prevent subsequent addition of monomeric peptide. Hydrophobic interactions have been identified as one of the principal driving forces in the assembly of Aβ aggregates [19]. Serum albumin possesses multiple hydrophobic binding domains [20]. It is therefore reasonable to speculate that BSA may prevent the addition of monomer to fibrils by capping exposed hydrophobic sites. A similar hypothesis was proposed by Milojevic et al. to explain the arrest of monomer-oligomer exchange following the preferential binding of HSA to high molecular weight oligomers [13].

In solutions of newly formed aggregation intermediates, the ensuing effect of such strategic capping may be the trapping of small soluble aggregates that display a high physiological activity. Trapping of small aggregates has been observed for other molecules that inhibit Aβ assembly, including naphthalene sulfonates [21], inositol [22], calmidazolium chloride [23], an antibody recognizing Aβ_1–11 _[24], and submicellar concentrations of sodium dodecyl sulfate [25]. In the current study, both thioflavin T fluorescence and DLS were employed to illustrate that the relative ratio of inhibitor to monomeric Aβ_1–40 _determines both the quantity (Figure 1) and size (Figure 4) of aggregates trapped. As expected, uninhibited reactions allowed the formation of significant quantities of large Aβ_1–40 _aggregates and complete inhibition yielded primarily monomeric or oligomeric Aβ_1–40_. In contrast, intermediate levels of inhibition by BSA led to the accumulation of soluble aggregation intermediates. Moreover, this accumulation was paralleled by an increase in Aβ stimulation of endothelial monolayers for monocyte adhesion (Figure 3). Some extent of this observed stimulation might be simply explained by the kinetic reversibility of binding between BSA and Aβ_1–40 _aggregates. However, the accumulation of small aggregate species is likely responsible for the augmented stimulation of endothelial monolayers relative to that observed for Aβ_1–40 _aggregates prepared in the absence of BSA. Aβ aggregate size has been shown previously to correlate inversely with the ability of aggregates to stimulate endothelial monolayers for monocyte adhesion [6] and permeability [7]. These results further demonstrate that small aggregates formed as a result of trapping by an inhibitor also exhibit a higher physiological activity. Conversely, Pallitto et al. observed a decrease in neurotoxicity when Aβ_1–39 _was incubated in the presence of a small peptide capable of accelerating aggregation and increasing aggregate size [26].

## Conclusion

Results presented within this study support implications that small soluble Aβ aggregates may play a principal role in AD pathogenesis [reviewed in [2,5]] and emphasize the danger of extrapolating *in vitro *Aβ inhibition data to reductions in physiological activity. The trapping of small soluble aggregates by both endogenous inhibitors, such as serum albumin, as well as synthetic compounds can lead to an undesired increase in physiological activity. Such potential consequences will be critical to consider in the future design of therapeutic agents targeted at inhibition of Aβ assembly.

## Abbreviations

Aβ: amyloid-β peptide; AD: Alzheimer's disease; BSA: bovine serum albumin; D-PBS: Dulbecco's phosphate buffered saline; DLS: dynamic light scattering; FBS: fetal bovine serum; HSA: human serum albumin; HBMVEC: human brain microvascular endothelial cell; *R*_H_: hydrodynamic radius.

## Competing interests

The authors declare that they have no competing interests.

## Authors' contributions

AARB conducted Aβ monomer aggregation and fibril elongation studies, supplied Aβ aggregates for cell adhesion assays, and drafted the manuscript. FJGV conducted cell adhesion assays. MAM conceived of the study, participated in the design and coordination of the study, and helped to draft the manuscript. All authors read and approved the final manuscript.

## References

[B1] Alzheimer's Association (2008). 2008 Alzheimer's disease facts and figures. Alzheimers Dement.

[B2] Walsh DM, Selkoe DJ (2007). Aβ oligomers – A decade of discovery. J Neurochem.

[B3] Goedert M, Spillantini MG (2006). A century of Alzheimer's disease. Science.

[B4] Eriksen JL, Janus CG (2007). Plaques, tangles, and memory loss in mouse models of neurodegeneration. Behav Genet.

[B5] Kirkitadze MD, Bitan G, Teplow DB (2002). Paradigm shifts in Alzheimer's disease and other neurodegenerative disorders: The emerging role of oligomeric assemblies. J Neurosci Res.

[B6] Gonzalez-Velasquez FJ, Moss MA (2008). Soluble aggregates of the amyloid-β protein activate endothelial monolayers for adhesion and subsequent transmigration of monocyte cells. J Neurochem.

[B7] Gonzalez-Velasquez FJ, Kotarek JA, Moss MA (2008). Soluble aggregates of the amyloid-β protein selectively stimulate permeability in human brain microvascular endothelial monolayers. J Neurochem.

[B8] De Felice FG, Ferreira ST (2002). Physiopathological modulators of amyloid aggregation and novel pharmacological approaches in Alzheimer's disease. An Acad Bras Cienc.

[B9] Kuo YM, Kokjohn TA, Kalback W, Luehrs D, Galasko DR, Chevallier N, Koo EH, Emmerling MR, Roher AE (2000). Amyloid-β peptides interact with plasma proteins and erythrocytes: implications for their quantitation in plasma. Biochem Biophys Res Commun.

[B10] Biere AL, Ostaszewski B, Stimson ER, Hyman BT, Maggio JE, Selkoe DJ (1996). Amyloid beta-peptide is transported on lipoproteins and albumin in human plasma. J Biol Chem.

[B11] Morgan C, Colombres M, Nunez MT, Inestrosa NC (2004). Structure and function of amyloid in Alzheimer's disease. Prog Neurobiol.

[B12] Bohrmann B, Tjernberg L, Kuner P, Poli S, Levet-Trafit B, Naslund J, Richards G, Huber W, Dobeli H, Nordstedt C (1999). Endogenous proteins controlling amyloid β-peptide polymerization. Possible implications for β-amyloid formation in the central nervous system and in peripheral tissues. J Biol Chem.

[B13] Milojevic J, Esposito V, Das R, Melacini G (2007). Understanding the molecular basis for the inhibition of the Alzheimer's Aβ-peptide oligomerization by human serum albumin using saturation transfer difference and off-resonance relaxation NMR spectroscopy. J Am Chem Soc.

[B14] Necula M, Kayed R, Milton S, Glabe CG (2007). Small molecule inhibitors of aggregation indicate that amyloid β oligomerization and fibrillization pathways are independent and distinct. J Biol Chem.

[B15] Giri R, Shen Y, Stins M, Du Yan S, Schmidt AM, Stern D, Kim KS, Zlokovic B, Kalra VK (2000). β-amyloid-induced migration of monocytes across human brain endothelial cells involves RAGE and PECAM-1. Am J Physiol Cell Physiol.

[B16] Giri R, Selvaraj S, Miller CA, Hofman F, Yan SD, Stern D, Zlokovic BV, Kalra VK (2002). Effect of endothelial cell polarity on β-amyloid-induced migration of monocytes across normal and AD endothelium. Am J Physiol Cell Physiol.

[B17] Gonzalo-Ruiz A, Perez JL, Sanz JM, Geula C, Arevalo J (2006). Effects of lipids and aging on the neurotoxicity and neuronal loss caused by intracerebral injections of the amyloid-β peptide in the rat. Exp Neurol.

[B18] Rozga M, Kloniecki M, Jablonowska A, Dadlez M, Bal W (2007). The binding constant for amyloid Aβ40 peptide interaction with human serum albumin. Biochem Biophys Res Commun.

[B19] Lin MS, Chen LY, Tsai HT, Wang SS, Chang Y, Higuchi A, Chen WY (2008). Investigation of the mechanism of β-amyloid fibril formation by kinetic and thermodynamic analyses. Langmuir.

[B20] Sugio S, Kashima A, Mochizuki S, Noda M, Kobayashi K (1999). Crystal structure of human serum albumin at 2.5 A resolution. Protein Eng.

[B21] Ferrao-Gonzales AD, Robbs BK, Moreau VH, Ferreira A, Juliano L, Valente AP, Almeida FC, Silva JL, Foguel D (2005). Controlling β-amyloid oligomerization by the use of naphthalene sulfonates: Trapping low molecular weight oligomeric species. J Biol Chem.

[B22] McLaurin J, Golomb R, Jurewicz A, Antel JP, Fraser PE (2000). Inositol stereoisomers stabilize an oligomeric aggregate of Alzheimer amyloid β peptide and inhibit Aβ-induced toxicity. J Biol Chem.

[B23] Williams AD, Sega M, Chen M, Kheterpal I, Geva M, Berthelier V, Kaleta DT, Cook KD, Wetzel R (2005). Structural properties of Aβ protofibrils stabilized by a small molecule. Proc Natl Acad Sci USA.

[B24] Mamikonyan G, Necula M, Mkrtichyan M, Ghochikyan A, Petrushina I, Movsesyan N, Mina E, Kiyatkin A, Glabe CG, Cribbs DH, Agadjanyan MG (2007). Anti-Aβ_1–11 _antibody binds to different beta-amyloid species, inhibits fibril formation, and disaggregates preformed fibrils but not the most toxic oligomers. J Biol Chem.

[B25] Tew DJ, Bottomley SP, Smith DP, Ciccotosto GD, Babon J, Hinds MG, Masters CL, Cappai R, Barnham KJ (2008). Stabilization of neurotoxic soluble β-sheet-rich conformations of the Alzheimer's disease amyloid-β peptide. Biophys J.

[B26] Pallitto MM, Ghanta J, Heinzelman P, Kiessling LL, Murphy RM (1999). Recognition sequence design for peptidyl modulators of β-amyloid aggregation and toxicity. Biochemistry.

